# Factor Analysis for Bicluster Acquisition (FABIA) revealed vincristine-sensitive transcript pattern of canine transmissible venereal tumors

**DOI:** 10.1016/j.heliyon.2019.e01558

**Published:** 2019-05-14

**Authors:** K. Chokeshaiusaha, D. Puthier, C. Nguyen, P. Sudjaidee, T. Sananmuang

**Affiliations:** a Department of Veterinary Science, Faculty of Veterinary Medicine, Rajamangala University of Technology Tawan-OK, Chonburi, Thailand; b Aix Marseille Univ, TAGC INSERM UMR 1090, Marseille, France

**Keywords:** Cell biology, Immunology

## Abstract

Chemotherapeutic treatment for Canine transmissible venereal tumor (CTVT) commonly relies on vincristine administration. Since the treatment outcomes can vary among CTVT cases, gaining insight into the tumor cell mechanisms influencing vincristine's potency should render veterinarians novel knowledge to enhance its therapeutic effect. This study aimed to attain such knowledge from a meta-analysis of CTVT mRNA sequencing (mRNA-seq) transcriptome data using Factor Analysis for Bicluster Acquisition (FABIA) biclustering.

FABIA biclustering identified 459 genes consistently expressed among mRNA-seq transcription profiling of CTVT samples regressed by vincristine. These genes were also differentially expressed from those of progressive CTVT (FDR ≤ 0.001). Enrichment analysis illustrated the affiliation of these genes with “Antigen presentation” and “Lysosome” GO terms (FDR ≤ 0.05). Several genes in “Lysosome” term involved 5 cell mechanisms—antigen presentation, autophagy, cell-adhesion, lysosomal membrane permeabilization (LMP), and PI3K/mTOR signaling. This study integrated FABIA biclustering in CTVT transcriptome analysis to gain insight into cell mechanisms responsible for vincristine-sensitive characteristics of the tumor, in order to identify new molecular targets augmenting therapeutic effect of vincristine. Interestingly, the analysis indicated LMP targeting by lysosome destabilizing agent—siramesine as the promising vincristine's enhancer for future study. As far as we know, this is the first canine tumor transcriptomic meta-analysis applying FABIA biclustering for the betterment of future CTVT therapy. This study hereby provided an interesting manifestation to acquire such knowledge in other canine neoplasia.

## 1 Introduction

Canine transmissible venereal tumor (CTVT) is a unique contagious canine neoplasia transmissible via mucosal contact and abrasions as allograft (Das and Das, 2000). CTVT progression is indexed by its growth rate along with its histopathological feature (Birhan and Chanie, 2015; Islam et al., 2017). In progressive phrase, CTVT mass grows rapidly within a week bestowing active round tumor cells showing significant diffused hyperchromatic nuclei and mitotic figures. In response to effective treatment or immunity, CTVT would attain stable phrase impairing its growth rate from weeks to months. Finally, it would manifest marked regression in the regressive phrase, during which the active tumor cells were evanescent (Islam et al., 2017; Park et al., 2006).

Most CTVT cases are progressive with undefined tumor mechanisms (Birhan and Chanie, 2015; Frampton et al., 2018; Siddle and Kaufman, 2015). In most human tumors, aberrant control of several cell mechanisms were acknowledged during tumor progression. Some of these mechanisms, for instance, cell adhesion (Besson et al., 2008; Li, 2008), autophagy (Crotzer and Blum, 2009), cyclin-dependent kinase (CDK) inhibtion (Besson et al., 2008), and lysosomal membrane permeabilization (LMP)-induced cell death (Groth-Pedersen et al., 2007; Serrano-Puebla and Boya, 2018) were recently acclaimed as novel targets for tumor therapy. On the contrary, the tumor feedback mechanisms to some well-recognized tumor drugs, such as, phosphatidylinositol-3-kinase (PI3K) and the mammalian target of rapamycin (mTOR)—the PI3K/mTOR signaling inhibitors (Stratikopoulos and Parsons, 2016), and resistance to anti-mitotic agent (Birhan and Chanie, 2015; Report et al., 2012) currently became the critical therapeutic issues. While contributing roles of these cell mechanisms on CTVT progression were limitedly acknowledged, gaining insight into them should provide us clues for the betterment of future CTVT therapy.

Vincristine sulfate is the well-known anti-mitotic agent of choice against CTVT (Das and Das, 2000; Report et al., 2012). By binding to tubulin spindle fiber, vincristine can arrest tumor chromosome segregation at metaphase stage (Jordan, 2012). In human neoplasia, vincristine-induced cell death was additionally evidenced to affiliate with LMP-induced cell death (Groth-Pedersen et al., 2007; Serrano-Puebla and Boya, 2018). However, such mechanism was not clearly verified in CTVT. Despite its efficiency, evidences of CTVT resistant cases were accumulated. This emphasized the necessity to understand CTVT resistant mechanisms for better therapeutic regimens to cope with them (Das and Das, 2000; Report et al., 2012; Rogers et al., 1998). Interestingly, recent advance in CTVT mRNA sequencing provided us an opportunity to gain insight into such cryptic mechanisms (Frampton et al., 2018).

Tumors in different phrases render different transcription profiles distinct from one another. It is hereby supportive of CTVT regressed by vincristine to demonstrate unique transcription patterns of mRNA sequencing data and vice versa. In order to discover such transcript patterns, Factor analysis for bicluster acquisition—FABIA was considered as a powerful data mining technique to extract such knowledge from transcriptome data (Eren et al., 2013). FABIA allowed simultaneous clustering of both tumor samples and their transcripts even with presence of minor differences, and thus was able to capture the correlated transcripts among undefined sample subsets with small bias (Eren et al., 2013; Hochreiter et al., 2010). Such co-clustering of sample subsets and gene transcripts—the biclusters could point out even the small changes in biological pathways modulated among tumor cells in different conditions (Besson et al., 2008; Skonieczna et al., 2017; Stratikopoulos and Parsons, 2016). Despite its absence of application in CTVT transcriptome studies, FABIA should be effective in identifying the vincristine-responsive transcript patterns of CTVT. By means of this, the acquired biclustering genes would further imply tumor cell mechanisms which could hint us novel approach to induce CTVT regression.

In this study, FABIA biclusters of vincristine-responsive CTVT transcription profiles were identified. Several of these genes encoded lysosomal proteins—of which their functions were associated with particular cell mechanisms and their vincristine-sensitive features. Unlike the previous study which concentrated on describing tumor genetic background and host-tumor interaction (Frampton et al., 2018), this study aimed to gain insight into noteworthy cell mechanisms responsible for vincristine-induced CTVT regression. With the acquired knowledge, novel chemotherapeutic approaches were introduced providing exiting several open-topics for future studies of CTVT therapy.

## 2 Materials and methods

### 2.1 Sample datasets

List of CTVT mRNA sequencing datasets used in this study were generated from NextSeq sequencing technology, and were freely available in Sequence Read Archive (SRA) database (https://www.ncbi.nlm.nih.gov/sra). The SRA datasets and their descriptions were provided (Table 1). In brief, CTVT samples used for dataset generation were acquired 3 tumor phrases—regressive, progressive and stationary phrases. Of note, all CTVT samples in regressive phrase were obtained after vincristine treatment. Further details of each dataset could be achieved directly from SRA database.

**Table 1:** CTVT datasets used in this study.

Datasets	Phrase of CTVT biosample	Day after vincristine treatment (0.025 mg/kg)
ERR1846288	Regressive	Day 14
ERR1846291	Regressive	Day 14
ERR2044814	Regressive	Day 28
ERR2044816	Regressive	Day 28
ERR2044818	Regressive	Day 28
ERR1846287	Progressive	None
ERR1846290	Progressive	None
ERR2044811	Progressive	None
ERR2044812	Progressive	Day 28
ERR2044813	Progressive	None
ERR2044815	Progressive	None
ERR2044817	Progressive	None
ERR2044819	Progressive	None
ERR2044820	Progressive	Day 28
ERR2044821	Progressive	None
ERR2044822	Progressive	Day 28
ERR1846289	Stationary	Day 6
ERR1846292	Stationary	Day 6

### 2.2 Data preprocessing

The Sequence Read Archive (SRA) files of CTVT mRNA sequencing datasets were retrieved from SRA database (https://www.ncbi.nlm.nih.gov/sra) (Table 1), subsequently extracted, and pre-processed similar to those previously described (Chokeshaiusaha et al., 2018a, 2018b, 2016) In brief, sequence trimming was performed by Cutadapt program to remove contaminated adapter sequences and unqualified sequences. Only sequences with length ≥ 25 nucleotides and mean Phred score ≥ 25 were selected for canine genome—CanFam3.1 alignment by STAR software. Duplicated sequences were remove and corrected for GC base balance. Gene-level counting was performed by HTSeq software following by regularized-logarithm transformation and batch correction to normalize all count read datasets.

### 2.3 Differential biclustering genes

Factor analysis for bicluster acquisition (FABIA) was performed with the pre-processed CTVT datasets using “fabia” package (Hochreiter et al., 2010). The expected bicluster numbers ranged from 1 to 3 biclusters according to the number of CTVT phrases, and only biclusters containing with all tumor samples of their phrases would be considered (1,000 iterations and *α* ≤ 0.01). Gene-level deferential expression analysis between regressive and progressive CTVTs was performed by DESeq2 package (FDR ≤ 0.001 and log2-fold-change ≥ 2) (Love et al., 2014). Only differentially expressed genes presented in the acquired biclusters would be considered and included in gene-annotation enrichment analysis.

### 2.4 Gene-annotation enrichment analysis

Functional properties of the acquired differential biclustering genes were evaluated by gene-annotation enrichment analysis using ‘goseq’ package (Young et al., 2010). By available human-dog orthologous genes, significant Gene Ontology (GO) terms of both species were extracted from GO consortium database (FDR ≤ 0.05).

### 2.5 Data virtualization

Principal Component Analysis (PCA) was performed on pre-processed datasets and plot for virtualization in 3-dimensions as previously described (Chokeshaiusaha et al., 2016). Circular and combined heatmaps were drawn by ‘circlize’ (Gu et al., 2014) and ‘ComplexHeatmap’ packages (Gu et al., 2016), respectively. To present significant canine and human Gene Ontology—GO terms pertained from enrichment analysis, the word clouds were generated using ‘Gosummaries’ package (Kolde and Vilo, 2015).

## 3 Results

### 3.1 FABIA biclustering was able to discover unique gene expression patterns of regressive CTVT samples

**Fig. 1:**
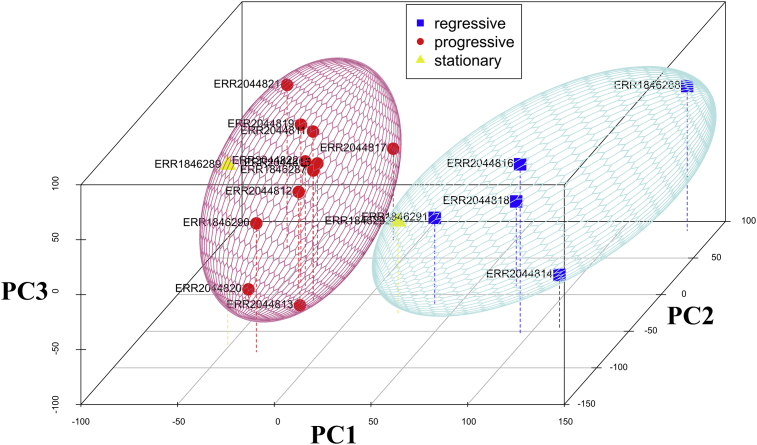
The Principle Component Analysis (PCA) results of CTVT samples were presented in 3-dimensional plot. The x, y and z axes represented the principal component 1 (PC1), principal component 3 (PC3), and principal component (PC2) values, accordingly. The blue rectangles, red circles, and yellow triangles respectively represented regressive, progressive, and stationary CTVT samples. Clusters of regressive CTVT (blue sphere) and progressive CTVT (red sphere) were presented, while stationary CTVT samples seemed to scatter between the two clusters.

**Fig. 2:**
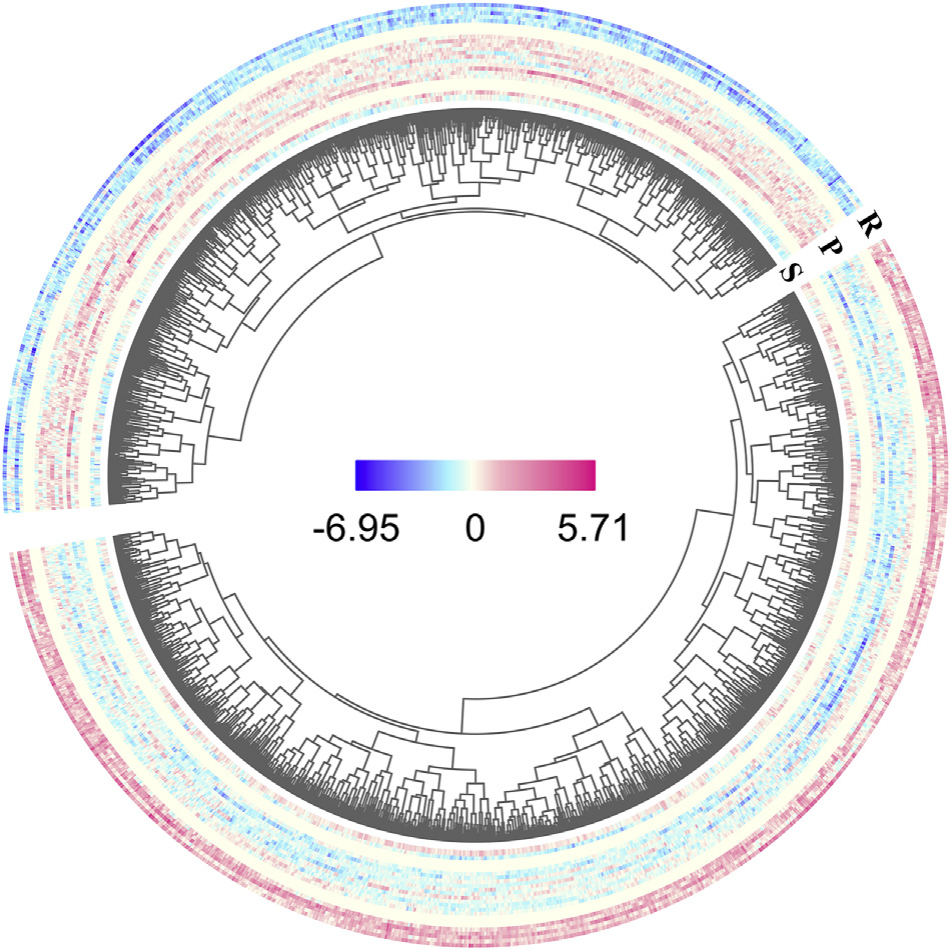
The circular heatmap demonstrated differential FABIA biclustering genes (columns) of CTVT samples (rows) grouped by phrases—R for regressive, P for progressive, and S for stationary phrases, accordingly. The genes were divided into 2 groups, low and high expression groups—according to their expression levels in regressive CTVT.

Principal Component Analysis (PCA) revealed separate clusters of regressive and progressive CTVT samples with stationary CTVT samples scattered among them—implying the differences in nature of CTVT transcriptome between regressive and progressive phrases. On the contrary, clustering of stationary CTVT samples was not presented (Fig. 1). Interestingly, FABIA biclustering successfully provided a solid bicluster containing overall regressive CTVT samples. Among expressed genes presented in the bicluster of regressive CTVT, 459 genes were found differentially expressed from those of progressive CTVT samples (FDR ≤ 0.001 and log2-fold-change ≥ 2). We did not include stationary CTVT in the analysis due to their inadequate sample numbers. These selected genes could be clearly classified into high and low expression groups concerning their relative expression levels in regressive CTVT (Fig. 2).

### 3.2 Enrichment analysis of acquired differential biclustering genes revealed “Antigen presentation” and “Lysosome” terms strongly associated with the transcript pattern of regressive CTVT

**Fig. 3:**
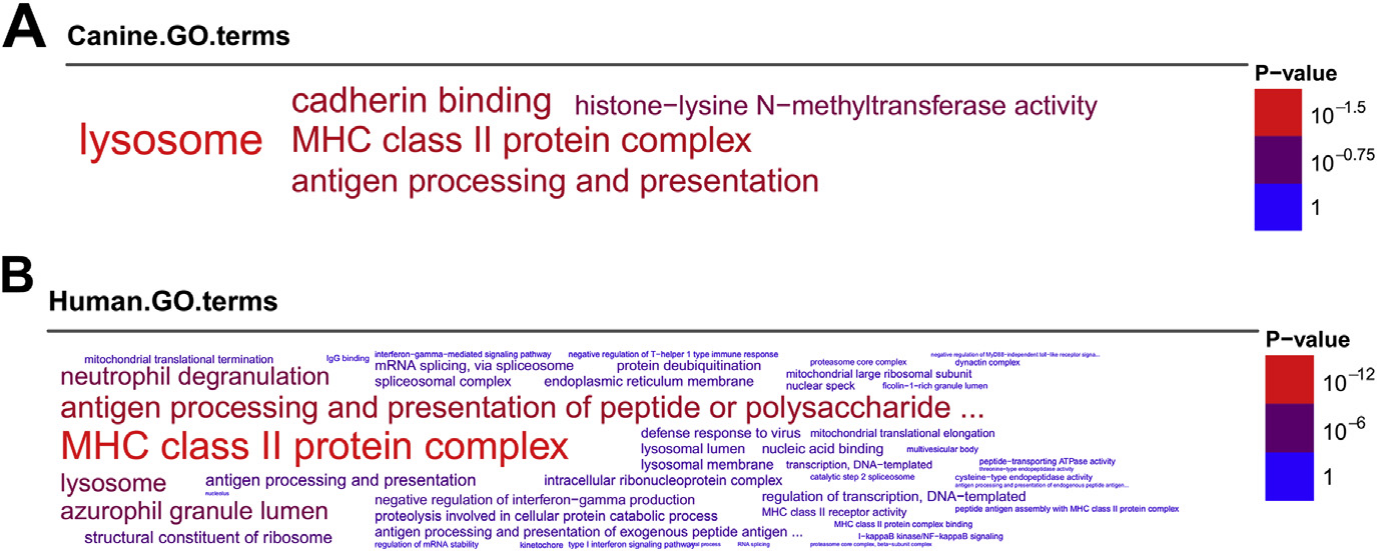
The word-clouds of canine (3A) and human (3B) GO annotation terms were generated from the differential biclustering genes. The term labels were plotted with their sizes proportional to numbers of genes presented, and with their colors ranged by their FDR values acquired from enrichment analysis.

**Fig. 4:**
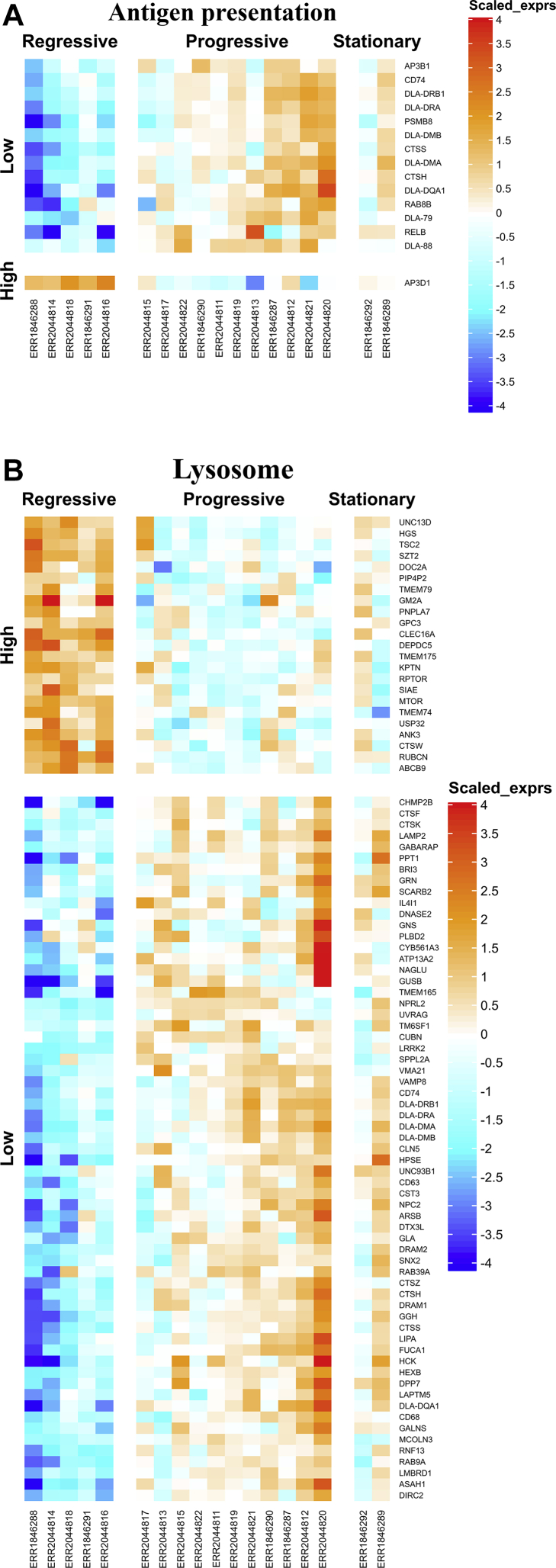
The heatmap of differential biclustering genes presented in “Antigen presentation” (4A) and “Lysosome” (4B) GO terms were illustrated. The heatmap row represented genes clustered by Pearson correlation distance with complete linkage, while the column represented CTVT samples categorized by their phrases.

Gene-annotation enrichment analysis revealed mutual significant terms achieved from both canine and human GO consortium databases, especially the 3 common terms—antigen processing and presentation, MHC class II protein complex (Fig. 3A), and lysosome (Fig. 3B) (FDR ≤ *0.05*). All genes presented in the “MHC class II protein complex” term were also in the “Antigen processing and presentation”, by which several genes were diminished in regressive CTVT, especially the canine MHC class II genes—the Dog Leukocyte Antigen (DLA) class II genes—DLA-DQA1, DLA-DMA, DLA-DRA, DLA-DMB (Fig. 4A). In addition, expressions of 2 DLA class I genes—DLA-79, and DLA-88 were also low in regressive CTVT. Interestingly, homogeneous expressions of numerous genes presented in the lysosome term were also manifested in regressive CTVT (FDR ≤ *0.05*) (Fig. 4B). While most of these genes were directly concerned with lysosomal function, several of them linked to other tumor cell mechanisms and features, by which would be further described in details.

Numbers of enzyme-encoding genes—ARSB, ASAH1, DPP7, DNASE2, FUCA1, GGH, GLA, GUSB, HEXB, IL4I1, LIPA, and PPT1, along with lysosome marker gene—TM6SF1 were lesser expressed in regressive CTVT despite some minor exceptions—SIAE and PNPLA7. These enzymes functioned in endosome, lysosome, phagosome, and autophagosome during autophagy (Anding and Baehrecke, 2017; Crotzer and Blum, 2009; Pasquier, 2016; Pearson and Soleimanpour, 2018). Expression heparan sulfate degrading-enzymes—GNS, HPSE, NAGLU were also diminished, while expressions of lysosomal membrane permeabilized proteases in cathepsin family—CST3, CTSF, CTSH, CTSK, CTSS, CTSW and CTSZ were varied.

Not only the enzyme-encoding genes, other genes in the “lysosome” term with indicated the functions related with some crucial cell mechanisms were also noticeable. These included PI3K/mTOR-associated genes—LRRK2, NPRL2, DEPDC5, TSC2, HCK, HGS, KPTN, RPTOR, RUBCN, SZT2 and MTOR; sorting/transpoort-protein genes—CHMP2B, CLN5, DOC2A, GM2A, LMBRD1, NPC2, SNX2, and TMEM79; autophagy-regulating genes— CHMP2B, GABARAP, LAMP2, MCOLN3, UVRAG, and VAMP8; and even antigen presentation-associated genes—ABCB9, CD74, and TMEM175.

### 3.3 Differential biclustering genes of regressive CTVT contained crucial genes associated with antigen presentation, autophagy, cell-adhesion, lysosomal membrane permeabilization (LMP), and PI3K/mTOR signaling

**Fig. 5:**
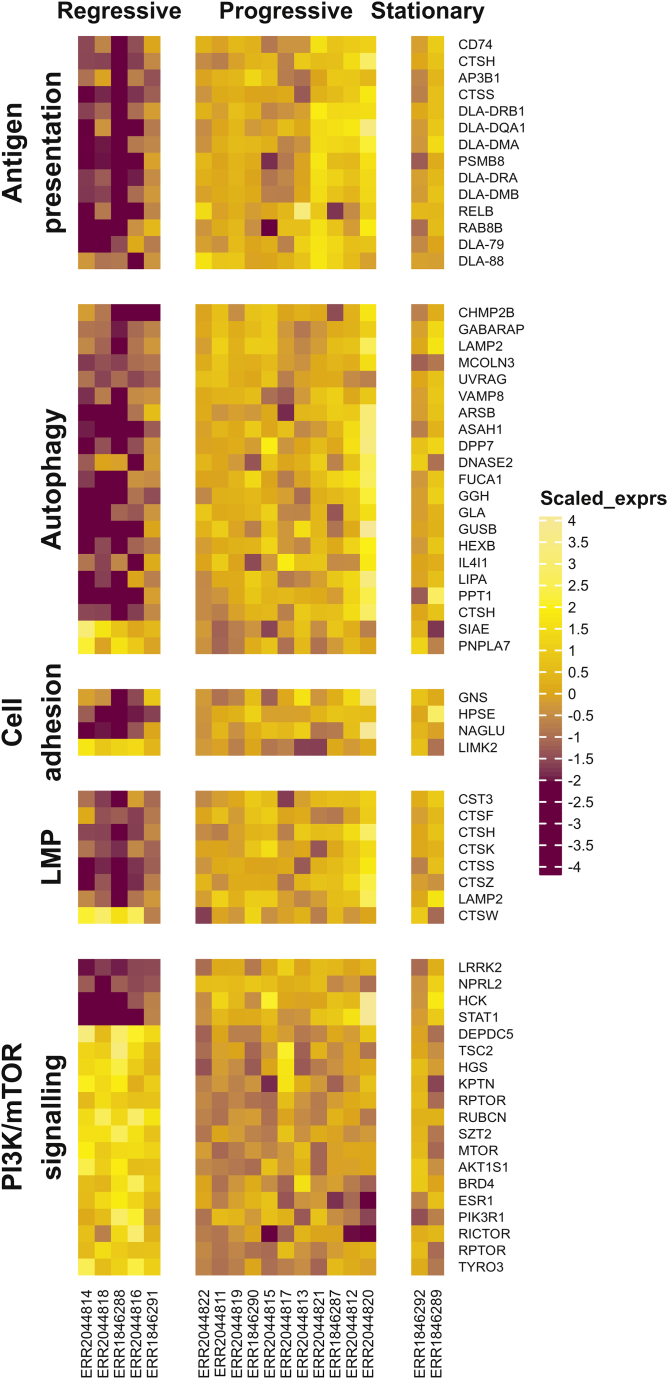
The heatmaps of the selected differential biclustering genes associated with 5 target cell mechanisms—Antigen presentation, Autophagy, Cell adhesion, Lysosomal membrane permeabilization (LMP), PI3K/mTOR signaling were illustrated. The heatmap row represented genes clustered by Pearson correlation distance with complete linkage, while the column represented CTVT samples categorized by their phrases.

According to the previous result, categorization of differential biclustering genes in the “lysosome” term suggested 5 major cell mechanisms related with biclustering genes of regressive CTVT. These cell mechanisms were as follows—antigen presentation, autophagy, cell-adhesion, lysosomal membrane permeabilization (LMP), and PI3K/mTOR signaling. We hereby included more differential biclustering genes with considerable roles in such mechanisms additional to those presented in lysosome term. While some genes were associated with several terms, we categorized them accordingly for simplicity. The overall genes, including those presented in lysosome term were categorized and separately demonstrated for convenient inspection (Fig. 5). Most of additional genes were associated with PI3K/mTOR pathway, and were expressed in regressive CTVT with significant levels—AKT1S1, BRD4, ESR1, PIK3R1, RICTOR, RPTOR, and TYRO3 (except for STAT1) (Fig. 5). Regressive CTVT also expressed significant LIMK2—an actin filament controlling gene affecting cell-to-cell adhesion (Fig. 5).

## 4 Discussion

Redundant cell mechanisms are common among tumor cells despite their comparable phrases or stages (Lavi, 2015). Varied genome-wide transcription patterns among CTVT samples even in the same phrase were thus common (Fig. 1). It was hereby reasonable that unique cell mechanisms modulated in each CTVT phrase should be denoted by its homogeneous transcript pattern unique from other phrases. In this study, FABIA biclustering captured such pattern in vincristine-responsive regressive CTVT (Fig. 2). Since such gene expression pattern reflected regressive CTVT's uniqueness, the cell mechanisms associated with them were, at certain degree, responsible for the tumor regression. Beneficially, such acquired knowledge should provide valuable insight into novel therapeutic agents targeting these cell mechanisms.

### 4.1 CTVT regressed from vincristine demonstrated low DLA class II antigen presentation

Vincristine-responsive CTVT was generally characterized by increased tumor cell apoptosis, along with reduced mitotic figures and reduced numbers of tumor infiltrating lymphocytes (TILs) (Mukaratirwa et al., 2009). Since IL-6 and IFN-γ secreted by TILs were major stimulants of DLA expression on CTVT cells (Hsiao et al., 2008), their absence should partially contribute to poor DLA gene expressions (Fig. 4A), and other genes associated with antigen processing, loading, and transportation (CTSS, CTSH, and PSMB8) (Fig. 5) in regressive CTVT. It should be however noted that, greater DLA class II gene expressions in progressive CTVT should bestow limited effects on spontaneous CTVT regression—which depended mainly on DLA class I antigen presentation to cytotoxic TILs (Hsiao et al., 2002; Mukaratirwa et al., 2009; Siddle and Kaufman, 2015). By mean of this, enhancing TIL function by recent immunotherapies, for instance by, autologous pulsed dendritic cell (Franco-Molina et al., 2018) or intratumoral IL-2 (Den Otter et al., 2015a, 2015b) could hereby provide putative synergistic effect with vincristine treatment.

### 4.2 Lysosomal membrane permeabilization (LMP) should be integrated into vincristine-induced CTVT regression

Vincristine was evidenced to sensitize lysosome's permeabilization in several human tumor cells (Groth-Pedersen et al., 2007; Serrano-Puebla and Boya, 2018). This resulted in leakage of several enzymes, especially specific cathepsins that could promote tumor cell death (Serrano-Puebla and Boya, 2018). Apart from varied expression of cathepsin-encoding genes (CST3, CTSF, CTSH, CTSK, CTSS, CTSW, and CTSZ), reduced lysosomal membrane integrity was also indicated in regressive CTVT (Fig. 5). To stabilize membrane integrity, Lysosome-associated membrane protein 2 (LAMP2) and Heatshock protein 70 (Hsp70) consolidated themselves into lysosomal membrane and functioned as major lysosomal membrane stabilizers (Groth-Pedersen et al., 2007; Serrano-Puebla and Boya, 2018). Poor expression of LAMP2 in regressive CTVT (Fig. 5) should hereby increased its sensitivity to LMP and thus susceptibility to vincristine, accordingly. Interestingly, the suggested LMP synergistic effect on vincristine endorsed the feasibility of lysosome destabilizing agents such as siramesine for CTVT therapy. Supporting such idea, siramesine was previously shown to synergize vincristine's effect on human breast tumor by reducing its dose-toxicity, and even overcoming the vincristine-resistant tumor cells (Groth-Pedersen et al., 2007). According to the evidence, we contemplated the favorable effects of siramesine on CTVT therapy, as well.

### 4.3 Impaired autophagy should contribute in CTVT's sensitivity to vincristine

Intracellular degradation of cytoplasmic proteins and organelles by autophagy is regarded as a fundamental cell mechanism for eliminating damaged proteins/organelles by lysosomal degradation (Anding and Baehrecke, 2017). Moreover, autophagy was even considered to provide particular antigens for MHC class II antigen presentation (Crotzer and Blum, 2009). Since removal of damaged proteins/organelles both prevented cell from such toxic byproducts and enabled recycle of nutrients (Anding and Baehrecke, 2017; Crotzer and Blum, 2009; Pasquier, 2016), autophagy should be considered as an crucial cell mechanism to protect CTVT cell from proteins/organelles damaged caused by vincristine (Groth-Pedersen et al., 2007).

Interestingly, differential biclustering genes strongly suggested inefficient autophagy in regressive CTVT. Supporting this notice, expressions of several enzyme-encoding genes required for degrading damaged molecules in lysosome (ARSB, ASAH1, DPP7, DNASE2, FUCA1, GGH, GLA, GUSB, HEXB, IL4I1, LIPA, and PPT1) were despicable. Furthermore, diminished expressions of regressive CTVT's transcripts were also observed in autophagy regulatory protein-encoding genes (UVRAG (Jo et al., 2018) and VAMP8 (Chen et al., 2015)), autophagosome sorting/channeling protein-encoding genes (CHMP2B (Krasniak and Ahmad, 2016), GABRAP (Nguyen et al., 2016) and MCOLN3 (Sun et al., 2016)) and even the gene encoding key receptor of chaperone-mediated autophagy—LAMP2 (Crotzer and Blum, 2009) (Fig. 5).

Since autophagy was required for both damaged protein clearance and antigen presentation, inefficient autophagy in regressive CTVT not only enhanced its susceptibility to vincristine-induced cell death, but also aggravated inhibition of DLA class II antigen presentation. Due to the supportive effect of autophagy suppression on vincristine treatment, we hereby anticipated its benefit on CTVT therapy by considering inclusion of autophagy inhibitors. However, it should be noted that, such agents could not provide exclusive targeting on cell autophagy (Pasquier, 2016), and thus were likely to cause integratively unpredictable effects when applied with CTVT.

### 4.4 Downstream inhibition of PI3K/mTOR signaling was suggested

PI3K/mTOR signaling is a complex cellular network regulating core cell mechanisms, such as, cell growth, cell proliferation and cell survival with signified crosstalks with other cellular signaling (Stratikopoulos and Parsons, 2016). PI3K/mTOR signaling is thus the major target for altering regulation in several types of tumors. In this study, downstream blockade of PI3K/mTOR signaling, albeit signs of increasing signaling molecules, was implied in CTVT regressed by vincristine. Increasing signaling molecules were suggested by high expression of PIK3R1 gene, which encoded p85α—the catalyzer of upstream signaling messenger—the phosphatidylinositol (3,4,5)-trisphosphate (PIP3) (Stratikopoulos and Parsons, 2016). Intensifying mTOR complex 1 and 2 (mTORC1 and mTORC2)—the well-known PI3K/mTOR mediators were also suggested due to enhanced expression of their component-encoding genes—RPTOR, RICTOR and MTOR genes (Earwaker et al., 2018; Sarbassov et al., 2005) (Fig. 5).

Despite the sign of increasing mediators, putative blockade in downstream signaling was presumed by high expressions of several mTORC1 inhibitor-encoding genes—DEPDC5 (Mizuno et al., 2018), KPTN (Saxton and Sabatini, 2017), NPRL2 (Saxton and Sabatini, 2017), SZT2 (Galluzzi et al., 2012; Wolfson et al., 2017), and TSC2 (Kang et al., 2011) (Fig. 5). Since mTORC1 was the key mediator of cell growth, overexpression of such inhibitors should result growth suppression of regressive CTVT (Stratikopoulos and Parsons, 2016). Additionally, other factors possibly contributing to signaling inhibition were also evidenced. These included enhanced expression of RUBCN—a PI3K inhibitor-encoding gene (Liu et al., 2017), limited expression of STAT1 mediator-encoding gene, limited expressions of ESR1 and TYRO3—the PI3K signaling receptor-encoding genes, and finally, the limited expressions of LRRK2 (Looyenga et al., 2011) and HCK (Poh et al., 2015)—the receptor associated kinase-encoding genes.

Implied mTORC1 signaling inhibition in vincristine-sensitive CTVT interestingly suggested certain PI3K/mTOR inhibitor—rapamycin as CTVT therapeutic agent. Rapamycin is a popular mTOR inhibitor applied among human tumor cases (Stratikopoulos and Parsons, 2016). Albeit the expected positive effects of combined vincristine and rapamycin on CTVT regression, the tumor feedback mechanisms against PI3K/mTOR inhibitors should also be regarded as possible drug-resistant complication (Stratikopoulos and Parsons, 2016).

### 4.5 Rigid cell adhesion was associated with regressive form of CTVT

Reduced adhesion of tumor cells to their adjacent cells and extracellular matrix (ECM) allow migration of progressive tumor (Besson et al., 2008; Report et al., 2012). For cell-to-cell adhesion, induction of actin stress fibers determines its strength (Sahai et al., 2001). Since LIM-kinase 2 mediated cell-to-cell adhesion via actin stress fiber assembly (Amano et al., 2001), high expression of its encoding gene—LIMK2 in regressive CTVT (Fig. 5) should thus impaired its progression. Additionally, regressive CTVT seemed to constitute higher adhesive strength to ECM than progressive CTVT, as well. ECM structure was majorly supported by arrays of heparan sulfate proteoglycan. These molecules not only strengthened cell adhesion, but also helped reserving factors required for tumor's blood vessel generation. Interestingly, several types of human tumors secreted heparanase to degrade heparansulfate—both for loosening ECM adhesion and releasing the trapped growth-promoting factors (Roy and Marchetti, 2009; Skonieczna et al., 2017). Low expressions of heparanase-encoding genes, especially HPSE, in regressive CTVT should hereby help limiting its progression (Fig. 5). By mean of this, administration of heparanase inhibitors, such as heparin, was likely to alleviate CTVT progression and metastasis.

## 5 Conclusions

Vincristine has been recognized as the drug of choice for CTVT therapy (Birhan and Chanie, 2015). Despite its long-term history of application, possible tumor cell mechanisms affecting its potency are still ambiguous. Differential biclustering genes in this study implied 4 cell mechanisms with consistent modulatory effects on vincristine's potency—LMP, autophagy, PI3K/mTOR signaling and cell adhesion. The acquired knowledge hereby established novel chemotherapeutic agents targeting such cell mechanisms to enhance therapeutic effect of vincristine. Owing to the suggested direct involvement of LMP with vincristine mechanism, we remarked the use of lysosome destabilizing agents, such as, siramesine as the most attractive approach. Given the success of vincristine-siramesine combination chemotherapy in human tumors (Groth-Pedersen et al., 2007; Serrano-Puebla and Boya, 2018), study of its sole efficiency on CTVT regression should be regarded as an exciting issue for further investigation. Of note, the current study manifested an original analytical procedure to screen for specific molecular features in canine neoplasia using FABIA method—by which similar approach could be achieved in other tumor studies.

## Declarations

### Author contribution statement

K Chokeshaiusaha: Conceived and designed the experiments; Performed the experiments; Analyzed and interpreted the data; Wrote the paper.

P Sudjaidee: Conceived and designed the experiments.

D Puthier: Performed the experiments; Analyzed and interpreted the data; Contributed reagents, materials, analysis tools or data.

C Nguyen: Contributed reagents, materials, analysis tools or data; Wrote the paper.

T Sananmuang: Conceived and designed the experiments; Contributed reagents, materials, analysis tools or data; Wrote the paper.

### Funding statement

This work was supported by Thailand Research Fund (TRF) through New Research Scholar Program (Grant No. TRG5880003).

### Competing interest statement

The authors declare no conflict of interest.

### Additional information

No additional information is available for this paper.
